# Culture-Free Phylogenetic Analysis of Legionella pneumophila Using Targeted CRISPR/Cas9 Next-Generation Sequencing

**DOI:** 10.1128/spectrum.00359-22

**Published:** 2022-07-11

**Authors:** Ana Domazetovska, Slade O. Jensen, Matthew Gray, Michael Radzieta, Michael Maley

**Affiliations:** a Department of Microbiology and Infectious Diseases, Liverpool Hospital, Liverpool, New South Wales, Australia; b NSW Health Pathology, Microbiology, Liverpool Hospital, Liverpool, New South Wales, Australia; c Infectious Diseases and Microbiology, School of Medicine, Western Sydney University, Sydney, Australia; d Antibiotic Resistance and Mobile Elements Group, Ingham Institute of Applied Medical Research, Sydney, Australia; University of California Davis

**Keywords:** CRISPR/Cas9, *Legionella pneumophila*, phylogenetic analysis, targeted NGS

## Abstract

Currently available methods for the laboratory investigation of Legionella pneumophila outbreaks require organism culture. The ability to sequence L. pneumophila directly from clinical samples would significantly reduce delays. Here, we develop a method for targeted next-generation sequencing (NGS) of selected L. pneumophila genes utilizing a CRISPR/Cas9-based target enrichment system. We determine the method’s utility by typing cultured L. pneumophila isolates and subsequently apply the method directly to patient samples. We sequenced 10 L. pneumophila isolates by 2 methods, (i) whole-genome sequencing (WGS) and (ii) targeted (CRISPR/Cas9-based) finding low-abundance sequences by hybridization (FLASH)-NGS, sequencing 57 selected genes. The targeted NGS of 57 genes was more efficient than WGS, and phylogenetic analysis of the 57 genes yielded the same classification of the L. pneumophila isolates as that based on analysis of whole-genome data. Furthermore, targeted NGS of L. pneumophila performed directly on patient respiratory samples correctly classified the patients according to their corresponding cultured isolates. This provides proof of concept that targeted NGS can be used to sequence L. pneumophila directly from patient samples. Studies on a larger number of patient samples will further validate this method. Nonetheless, CRISPR/Cas9 targeted NGS methods have the potential to be widely applicable to microbial-outbreak investigations in the future, particularly in the context of difficult and slow-growing organisms.

**IMPORTANCE** The bacterium Legionella pneumophila is responsible for outbreaks of serious and life-threatening pneumonia called Legionnaires’ disease. There is a need for new molecular methods that allow investigation of Legionella outbreaks directly from patient samples, without the need for prior microbiological culture, which causes delays. Our study aims to address this problem. We have utilized a CRISPR/Cas9-based targeted next-generation sequencing (NGS) method that can be applied directly on human specimens. Furthermore, we show that analysis of the sequences of a small number of targeted genes offers the same classification of L. pneumophila as that based on data derived from the whole genome. Given the rising interest globally in sequencing pathogens directly from human samples, CRISPR/Cas9 targeted NGS methods have the potential to be widely applicable to microbial-outbreak investigations in the future, particularly in the context of difficult and slow-growing organisms.

## INTRODUCTION

Legionella pneumophila is a cause of Legionnaires’ disease, a serious and life-threatening pneumonia. The inhalation of contaminated aerosols in the environment can lead to outbreaks of Legionnaires’ disease (LD) of public health significance. Timely typing of Legionella is important for outbreak investigation and management, but there is currently a lack of an accepted standard method for L. pneumophila typing. Over the past few years, with the wider availability and reduction in cost of next-generation sequencing (NGS), whole-genome sequencing (WGS) of Legionella isolates has become increasingly important in the investigation of point source outbreaks (1–5). Recently, it was reported that sequencing only a portion of the L. pneumophila genome (perhaps as few as 50 genes) could offer good discriminatory power and epidemiological concordance (6) and be as effective for outbreak investigation as the analysis of WGS data. However, most currently available options have their challenges, the most significant of which is the requirement for culture, which is slow and has poor sensitivity (7, 8).

Developing methods for direct sequencing of L. pneumophila from patient samples, without the requirement for culture, could have significant advantages in diagnosis and outbreak investigation. Obtaining whole-genome sequences by metagenomic next-generation sequencing (mNGS) directly from patient samples is difficult due to high levels of background human DNA and low target abundance. Methods like nested-PCR-based amplification of a smaller number of genes (7 genes) have been performed directly on patient samples (9–11); however, they have limited utility due to inferior discriminatory power compared to analysis of WGS data.

To enrich for low-abundance microbial nucleic acids from clinical samples, target enrichment methods (such as target capture or amplicon-based NGS approaches) (12) have been used to sequence the genomes of several pathogens, including severe acute respiratory syndrome coronavirus 2 (SARS-CoV-2) (13), Zika virus (14), Mycobacterium tuberculosis (15–17), and Neisseria meningitidis (18). Recently, a novel target enrichment method, finding low-abundance sequences by hybridization (FLASH)-NGS (19), which utilizes the CRISPR/Cas9 system, was applied to the detection of several thousand antimicrobial resistance genes in human respiratory samples. FLASH-NGS employs a set of small guide RNA molecules (CRISPR RNAs) that lead the Cas9 enzyme to cleave the target DNA into fragments appropriate for next-generation sequencing methods (19). The method is nonproprietary and thus freely accessible and has the potential to be widely applicable in clinical diagnostic microbiology.

Here, we have utilized FLASH-NGS for targeted sequencing of 57 genes and subsequent phylogenetic analysis of L. pneumophila. In this proof-of-principle study, we first tested the effectiveness of FLASH-NGS on bacterial isolates and then applied the method directly on human respiratory samples. The application of FLASH-NGS to Legionella typing will further demonstrate its versatility and pave the way for its wider use.

## RESULTS

### Validation of phylogenetic-analysis methods on a public data set of L. pneumophila genomes from cultured isolates.

We used a public data set of whole-genome sequence data from outbreak L. pneumophila isolates (Table S1 in the supplemental material) (5) to evaluate whether phylogenetic analysis based on the entire core-genome sequence (core-genome single-nucleotide polymorphism [SNP] phylogeny) could yield phylogeny congruent with that of analysis based on sequences from 57 genes (57-gene SNP phylogeny).

Analysis by both methods, core-genome SNP (Fig. S1A) and 57-gene SNP (Fig. S1B) phylogeny, based on 133,958 SNPs and 2,126 SNPs, respectively, produced results congruent with the published data (5) and confirmed phylogenetic relatedness between isolates that were epidemiologically associated with outbreaks in the Central Business District (CBD) and suburb 1 in Sydney in 2016 (Fig. S1, main cluster). Furthermore, the 2 methods were able to separate genomically distinct isolates not related to the main cluster, in agreement with published findings. These were isolates from cases GC1 to GC4 that were epidemiologically linked to suburb 2, along with case RC3 from suburb 1 and BC9/BC10 (2 isolates from same patient) with links to the CBD (Fig. S1).

### Assessing FLASH-NGS using cultured bacterial isolates of L. pneumophila.

**FIG 1:**
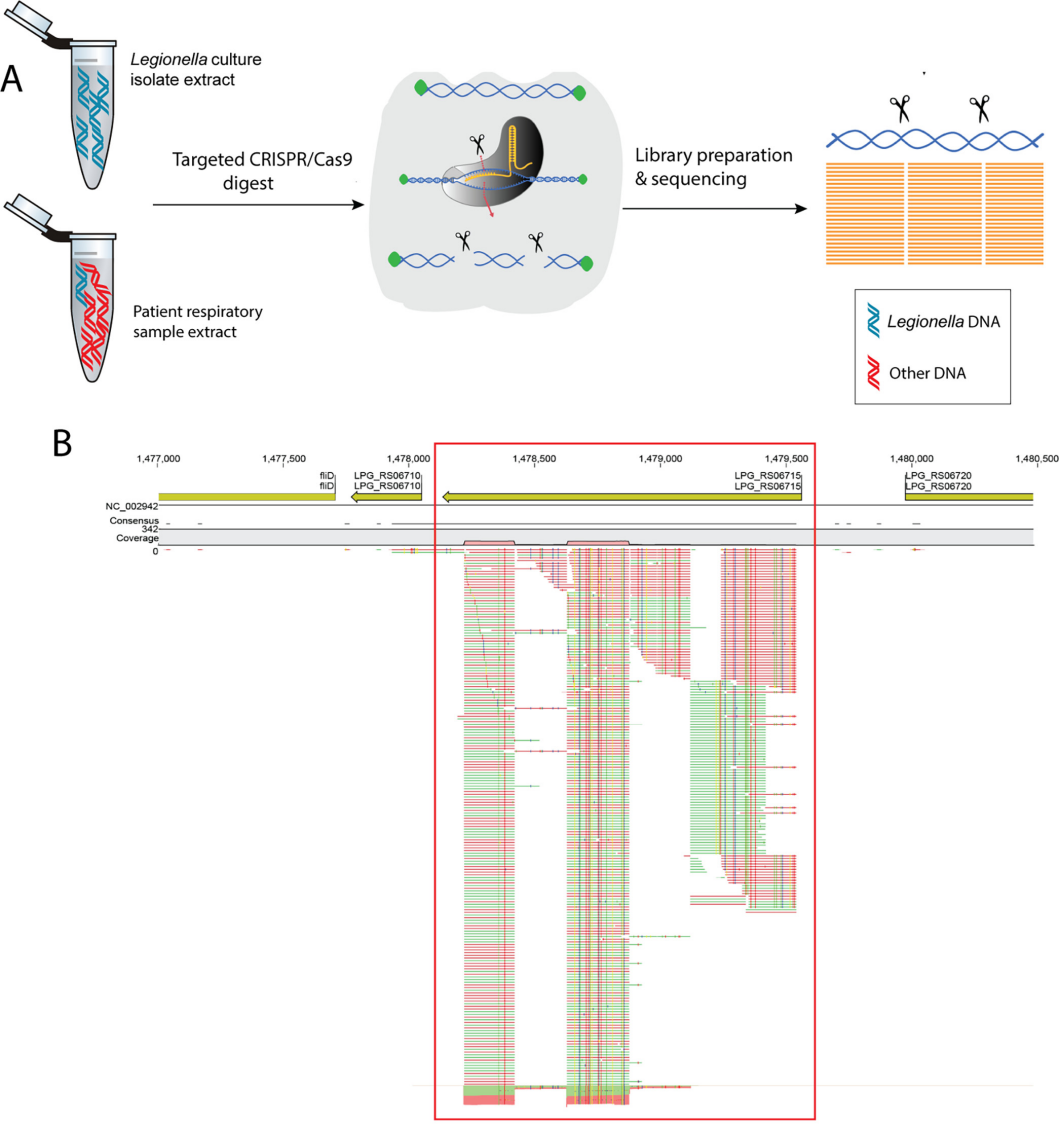

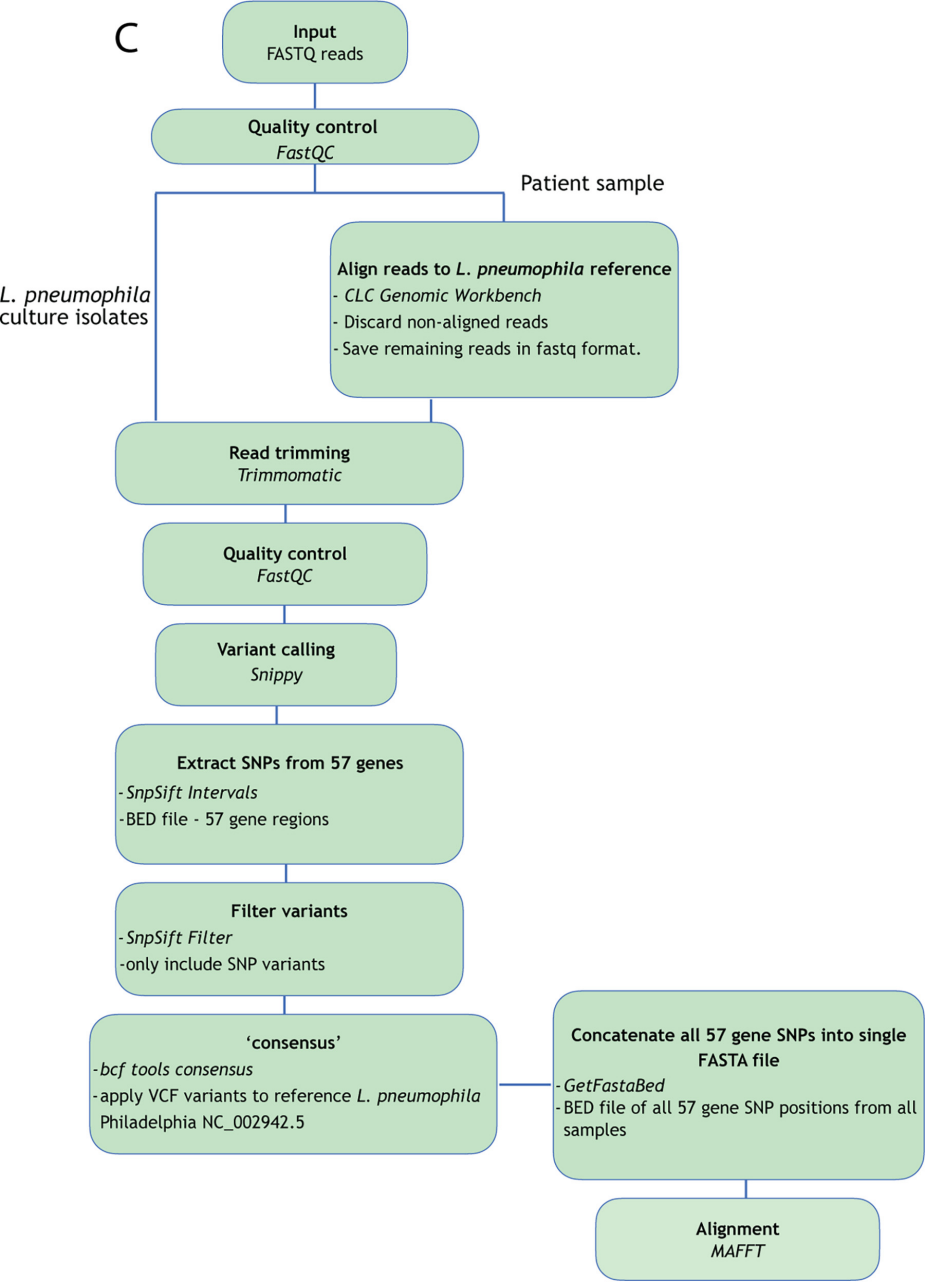
(A) Targeted sequencing method overview. Genomic DNA extracted from Legionella pneumophila isolates or patient samples is blocked by dephosphorylation (green circles). The CRISPR/Cas9 complex is guided to targeted sites in the genome by guide RNA, and the target DNA is digested by the Cas9 enzyme. Library preparation of the Cas9-digested DNA fragments and their sequencing follows. (B) Example of a gene targeted by FLASH-NGS (yellow arrow within red box) in the specimen from patient 1P. Sequence reads (red and green lines) are aligned to the targeted gene. There are no reads aligning to the nontargeted genes on either side. The image was generated using CLC Genomics Workbench. (C) Overview of the bioinformatics workflow for 57-gene SNP-based phylogenetic analysis. The main bioinformatics tools used for each task are mentioned in each box in italics. SNPs, single-nucleotide polymorphisms; BED, browser extensible data; VCF, variant call format.

**FIG 2:**
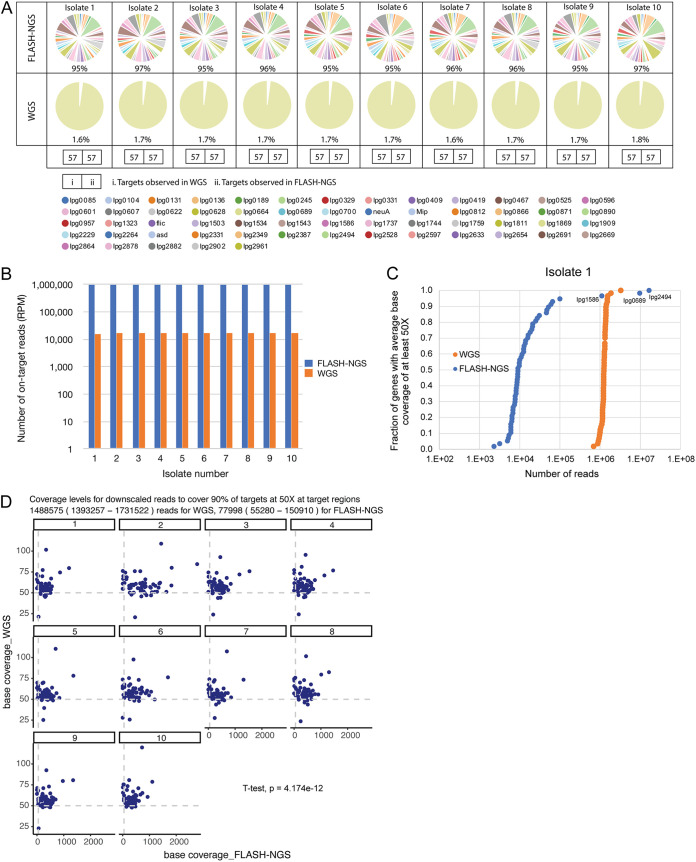
Results of FLASH-NGS of cultured L. pneumophila isolates. (A) Proportions of targeted genes detected in FLASH-NGS and WGS libraries of 10 L. pneumophila isolates. The numbers in boxes represent gene targets observed in WGS libraries (i) and gene targets observed in FLASH-NGS libraries (ii). The percentage of on-target reads is reported under each pie chart. The 57 target gene names are shown below the figure. (B) FLASH-NGS generated a 57-fold increase in average on-target reads per million (RPM) compared to WGS. (C) For isolate 1 with FLASH-NGS, 100,000 reads were needed to achieve average base coverage of the targeted genes of at least 50× (with the exception of 3 genes). Each data point represents a gene target. Over 1,000,000 reads were needed to achieve the same depth of base coverage with WGS. (D) A scaling factor was used to model coverage for the target regions for each isolate generated by FLASH-NGS and WGS. The required numbers of reads to cover at least 90% of target regions at 50× base coverage were 1,488,575 (1,393,257 to 1,731,522) for WGS and 77,998 (55,280 to 150,910) for FLASH-NGS (*P* = 4.174e−12, *t* test).

**FIG 3:**
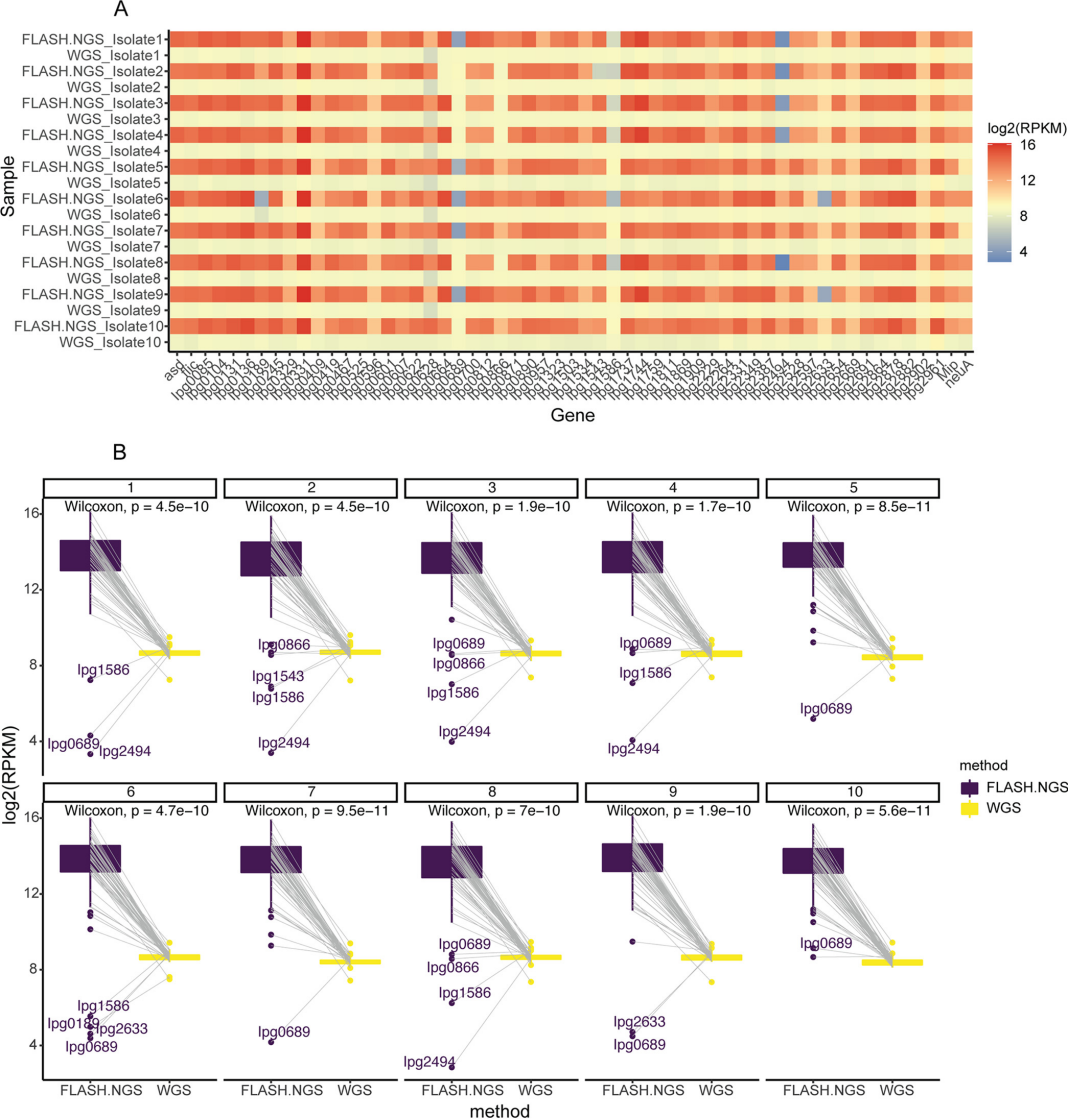
(A) Heatmap of the numbers of reads per kilobase per million (RPKM) aligning to 57 target genes generated by FLASH-NGS and WGS of 10 L. pneumophila isolates. (B) Paired Wilcoxon test shows that the target regions are significantly enriched with FLASH-NGS compared to the results for WGS.

The DNA extracted from 10 cultured isolates (Table 1) was sequenced by WGS and by FLASH-NGS(Fig. 1A) using 293 RNA guides targeting the 57 genes, and the results compared. All of the targeted genes were recovered in all 10 isolates by both methods (Fig. 2A). High coverage of nontargeted L. pneumophila regions did not occur with FLASH-NGS. On average, 95.7% of the total number of reads mapped to target genes for FLASH-NGS samples (Fig. 2A). In contrast, only 1.7% of total reads in WGS samples mapped to the 57 genes of interest (Fig. 2A). This represented a 57-fold increase in the average RPM (reads per million) obtained using FLASH-NGS compared to the average RPM obtained using WGS (Fig. 2B). For FLASH-NGS, 77,998 (55,280 to 150,910) reads were sufficient to cover 90% of target genes at 50× base coverage at target regions (Fig. 2C and D and Fig. S2). For WGS, 1,488,575 (1,393,257 to 1,731,522) reads were required to achieve the same depth of base coverage at the target regions. This was statistically significant (*P* = 4.174e−12, *t* test) (Fig. 2D). A heatmap comparing the numbers of reads per kilobase per million (RPKM) aligning to 57 target genes generated by FLASH-NGS and WGS is shown in Fig. 3A. The target genes were significantly enriched with FLASH-NGS compared to WGS (Fig. 3B), except for several genes discussed below.

**TABLE 1:** Legionella pneumophila isolates and patient samples included in this study

Isolate	Source	Epidemiological relationship^*a*^	Radiological evidence of pneumonia	*Legionella* PCR on respiratory sample	*Legionella* urine antigen	Corresponding clinical sample (patient, type of sample, *C_T_* value)^*b*^	ST^*c*^
01	Clinical isolate	Sporadic case (09/2019)	+	+	+	1P, ETT aspirate, 20	89
02	Clinical isolate	Sporadic case (07/2016)	+	+	+	2P, sputum, 35	84
03	Clinical isolate	Sporadic case (04/2018)	+	+	+	3P, sputum, 30	1694
04	Clinical isolate	Sporadic case (04/2018)	+	+	+	4P, sputum, 38	1694
05	Clinical isolate	Sporadic case (04/2018)	+	+	+	5P, sputum, 31	211
06	Clinical isolate	Sporadic case (10/2019)	+	+	+	NA	ND
07	Clinical isolate	Sporadic case (08/2019)	+	+	−	7P, ETT aspirate, 37	211
08	Clinical isolate	Sporadic case (03/2019)	+	+	+	NA	2437
09	Clinical isolate	Sporadic case (07/2016)	+	+	+	NA	ND
10	Control isolate (ATCC 33152)^*d*^						36

a Epidemiological relationship between cases before the molecular results; in sporadic cases, the month and year of the sample are included in brackets.

b ETT aspirate, endotracheal tube aspirate; NA, not available.

c ST, sequence type; ND, not determined due to one or more alleles being new or a combination of alleles being new.

d ATCC, American Type Culture Collection.

Of the 57 target genes, three genes (Ipg0689, Ipg1586, and Ipg2492) had consistently lower coverage in several isolates (Fig. 2B and Fig. S2). Correspondingly, these three genes were less abundant and had lower numbers of RPKM (<100) than the rest of the target genes, with an average RPKM of 17,525 (378 to 72,502) (Fig. 3B). Of interest, isolates 6 and 9 both had low coverage of gene Ipg2633 (Fig. S2), with RPKM values of <30 (Fig. 3B). This is a small gene of 203 bp with only 2 target sites for the CRISPR/Cas9 complex. Subsequently, the WGS data from this location were examined, and both isolates were found to harbor mutations in a target site, with one mutation located within the protospacer-adjacent motif (PAM) recognition site that is necessary for cleavage by the CRISPR/Cas9 complex (Fig. S3).

### Phylogenetic analysis of WGS and FLASH-NGS data from cultured L. pneumophila isolates.

**FIG 4:**
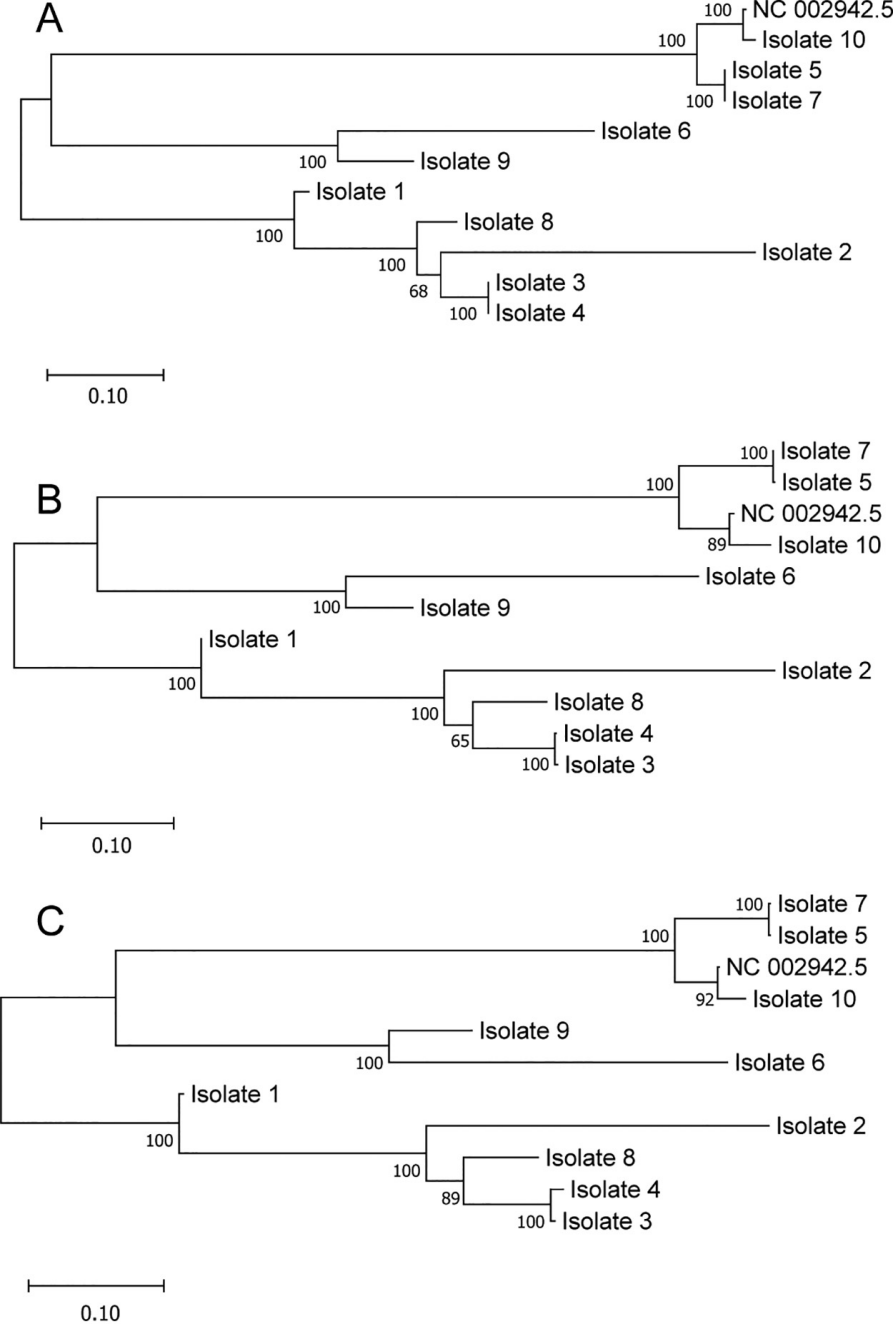
Phylogenetic analysis of WGS and FLASH-NGS data from 10 L. pneumophila isolates. (A) Core SNP-based mapping phylogeny of WGS data from 10 L. pneumophila isolates. A maximum-likelihood phylogenetic tree based on 127,175 core SNPs relative to the reference L. pneumophila Philadelphia genome sequence (NC_002942.5). The genomes of isolates 3 and 4 and isolates 5 and 7 were highly related. (B) The 57-gene SNP-based mapping phylogeny of the same WGS samples produced congruent results. Maximum-likelihood phylogenetic tree based on 1,922 SNPs within 57 genes. (C) The 57-gene SNP-based mapping phylogeny on FLASH-NGS data from 10 L. pneumophila isolates. Maximum-likelihood phylogenetic tree based on 1,830 SNPs within 57 genes relative to the reference L. pneumophila Philadelphia genome sequence (NC_002942.5). The phylogeny was congruent to that generated by analysis of WGS data.

Phylogenetic analysis of WGS data of the 10 isolates by core-SNP phylogeny and by 57-gene SNP phylogeny, based on 127,175 SNPs and 1,922 SNPs, respectively, yielded congruent results (Fig. 4A and B). Furthermore, phylogenetic analysis of sequence data obtained by FLASH-NGS of the 10 isolates using 57-gene SNP phylogeny (based on 1,830 SNPs) yielded the same classification of the isolates as analysis of WGS-NGS data (Fig. 4C). Two pairs of L. pneumophila genomes were found to be highly related (isolates 3 and 4 and isolates 5 and 7) by both core-genome SNP and 57-gene SNP phylogeny (Fig. 4), representing possibly epidemiologically linked cases. In addition, the genome of control isolate 10 (ATCC strain 33152) was, as expected, highly related to that of the L. pneumophila reference strain Philadelphia (GenBank accession NC_002942.5). These relationships were evident in both WGS and FLASH-NGS data.

### Assessing FLASH-NGS of L. pneumophila directly from patient samples.

**FIG 5:**
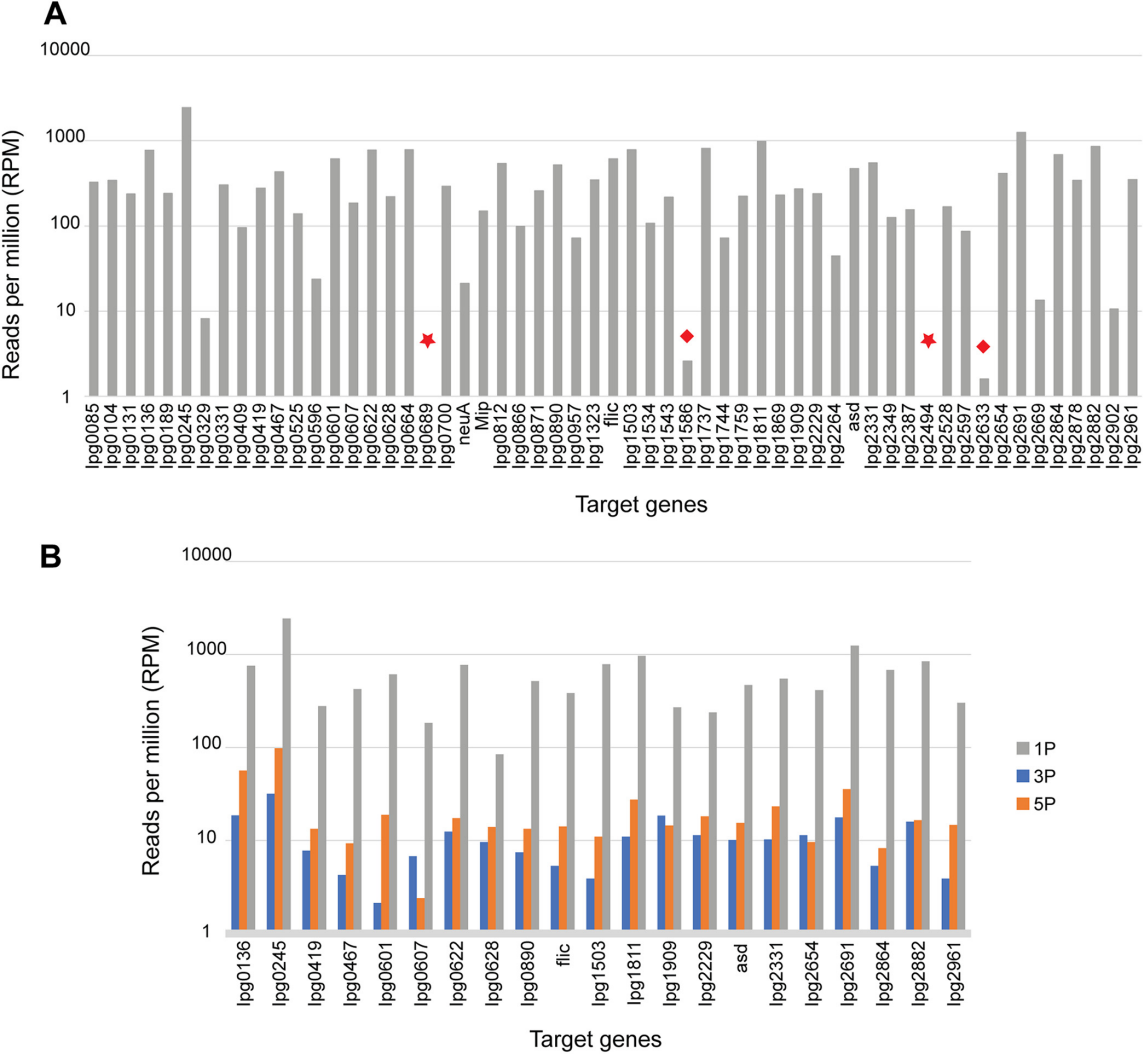
Results of FLASH-NGS of respiratory patient samples. (A) Numbers of reads per million aligning to 57 targeted genes detected by FLASH-NGS in DNA from respiratory sample from patient 1P. On-target reads were obtained for 55 of 57 targeted genes. No reads were obtained for 2 genes (stars), and 10 or fewer reads were obtained for another 2 genes (diamonds). (B) Numbers of reads per million aligning to fragments of 21 targeted genes detected by FLASH-NGS in DNA from respiratory samples from patients 1P, 3P, and 5P.

We attempted FLASH-NGS directly on respiratory tract samples from 6 patients who were hospitalized with culture-positive L. pneumophila pneumonia. These patients were designated 1P, 2P, 3P, 4P, 5P, and 7P and were the source of L. pneumophila isolates 1, 2, 3, 4, 5, and 7, respectively (Table 1). For patient 1P, FLASH-NGS on DNA extracted from an endotracheal tube (ETT) aspirate specimen yielded a total of 77,944 on-target reads for 55 of the 57 genes (Fig. 5A). For patients 3P and 5P, FLASH-NGS on DNA extracted from sputum samples yielded totals of 1,716 and 2,583 on-target reads, respectively, significantly less than for patient 1P (Fig. 5B). This corresponded to apparently smaller amounts of Legionella DNA in the samples, as supported by high Legionella PCR cycle threshold (*C_T_*) values for these patients’ samples compared to the *C_T_* value for the sample from patient 1P (30 and 31 versus 20) (Table 1). The samples from patients 2P, 4P, and 7P had even higher PCR *C_T_* values (35, 38 and 37), and hence, FLASH-NGS yielded only 197, 202, and 181 on-target reads, respectively, insufficient for further analysis.

Not all 57 targeted genes displayed adequate sequence coverage; for example, for the sample from patient 1P, approximately 10 reads were obtained for 2 genes (Ipg2633 and Ipg1586) and no reads were obtained for 2 genes (Ipg2494 and Ipg0689) (Fig. 5A). The latter 2 genes also had low coverage in the isolates. We found that of the 57 targeted Legionella genes, 34 regions within 21 genes had the highest overlapping coverage in the samples from patients 1P, 3P, and 5P, with 50,079, 1,272, and 1,692 reads, respectively (Fig. 5B and Fig. S4). Therefore, we used data from these 21 genes for phylogenetic analysis of the direct patient samples.

### Phylogenetic analysis of FLASH-NGS data direct from patient samples and from corresponding cultured isolates.

**FIG 6:**
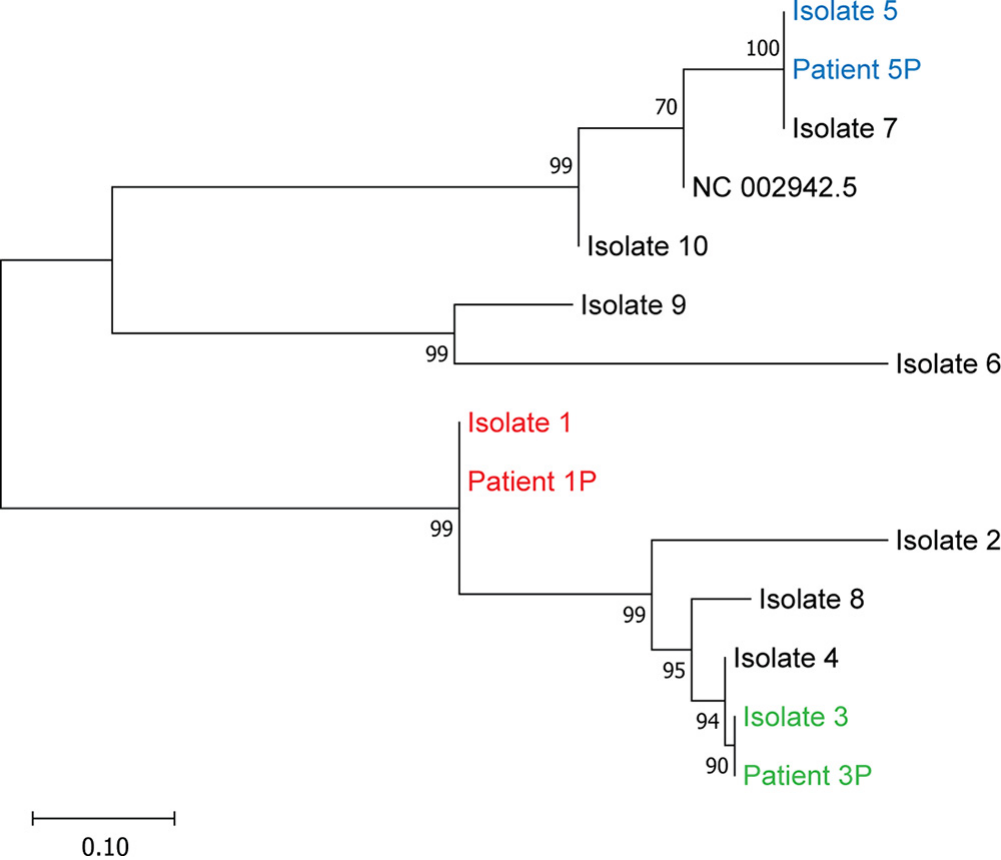
Phylogenetic analysis of FLASH-NGS data from patient respiratory samples and cultured L. pneumophila isolates using 21-gene-fragment SNP-based mapping phylogeny on the 10 L. pneumophila isolates and 3 samples from patients 1P, 3P, and 5P. Maximum-likelihood phylogenetic tree based on 320 SNPs within 21 genes relative to the reference genome sequence. The patient samples were identified as highly related to their corresponding isolates (matching colors of blue, red, or green).

For the sample from patient 1P (which had adequate coverage of 53 genes), 53-gene SNP phylogenetic analysis correctly identified the sample as highly related to isolate 1, with 100% similarity. Isolate 1 had been cultured from the same clinical sample and was expected to be identical (Fig. S5). Importantly, the removal of 4 genes from the analysis did not affect the overall phylogeny of the 10 isolates. We next compared phylogenetic analysis of sequence data covering the 34 regions within 21 genes that had the highest overlap for direct samples (1P, 3P, and 5P) and the 10 Legionella isolates. The 21-gene SNP phylogenetic analysis correctly identified the 3 patient samples as highly related to the corresponding cultured Legionella isolates 1, 3, and 5 (Fig. 6). The SNPs within the selected regions from 21 genes in samples 1P, 3P, and 5P were 100% identical to those of Legionella isolates 1, 3, and 5. Of importance, phylogenetic analysis based on 320 SNPs within 21 genes yielded the same phylogeny of L. pneumophila isolates as that based on analysis of 127,175 SNPs within the entire core genome (Fig. 6 and 4A, respectively). Furthermore, we additionally validated the 21-gene-fragment SNP phylogenetic analysis on the whole-genome sequence data from a public data set of outbreak L. pneumophila isolates (Fig. S1C).

## DISCUSSION

This study assesses the role of targeted next-generation sequencing in phylogenetic analysis of L. pneumophila. We demonstrate that FLASH-NGS can effectively target and enrich genes of interest using DNA from L. pneumophila isolates. Furthermore, we show that these selected genes can be used to correctly classify (via phylogenetic analysis of a public data set) related and unrelated L. pneumophila isolates. Finally, and of significant clinical importance, we show that FLASH-NGS can be used to accurately facilitate phylogenetic analysis of L. pneumophila directly from human respiratory samples, without prior culture.

One of the strengths of this study is the selection of a critical set of target core genes. The 57 selected genes showed substantial discriminatory power to categorize L. pneumophila into genomically related and unrelated strains with good epidemiological concordance. This is demonstrated by the 57-gene SNP phylogenetic analysis of the public data set, which correctly identified L. pneumophila isolates involved in the main cluster of the outbreak (Fig. S2). Similarly, in the 10 L. pneumophila isolates (Fig. 4), analysis of the 57 genes yielded the same phylogeny as analysis of SNPs of the entire core genome of over 1,500 genes. Note that 50 of the selected 57 genes have also been used in a core-genome multilocus sequence typing (cgMLST) scheme and were shown to be able to effectively resolve outbreak strains of L. pneumophila in Europe (6). Furthermore, we show that the analysis could be simplified to 21 genes, as demonstrated by the same phylogeny of 10 L. pneumophila isolates and 3 patient samples (Fig. 6) produced by the comparison of only 320 SNPs. Therefore, with the lack of a current “gold standard” for L. pneumophila typing, this study provides further support that phylogenetic analysis of about 50 core genes, or perhaps even as few as 21 genes, may be sufficient for the classification of L. pneumophila.

Targeted sequencing of L. pneumophila isolates by FLASH-NGS provides an alternative to WGS when focused analysis of key genes is required. For the 10 L. pneumophila isolates, FLASH-NGS led to more than a 50-fold increase in the number of on-target sequence reads compared to WGS (Fig. 2). Furthermore, FLASH-NGS required lower read numbers to achieve the same depth of coverage of the genes of interest as WGS. Due to only a portion of the genome being sequenced, this could lead to reduced sequencing costs and the generation of smaller and more manageable data sets than for WGS. At the time of conducting the experiments, the RNA guide preparation and CRISPR/Cas9 digest in this pilot experiment cost ~2,500 AUD with enough reagents for 50 samples. Even at this small scale, FLASH-NGS is a promising cost-saving strategy, considering the significant reduction in sequencing costs that could be achieved, although precise costing would need to account for the labor involved and possible automation of the process.

The main advantage of targeted FLASH-NGS is that it allows the enrichment of a large number of genes directly from human specimens. To the best of our knowledge, there are no reports of targeted NGS being used for Legionella typing, although direct WGS has been used in the genomic epidemiology of tuberculosis (17). As Legionella culture can take up to 14 days (7), the benefit of sequencing Legionella directly from clinical specimens is that it can significantly reduce time to result. Time is of vital importance, particularly with regard to outbreak investigation and control. The L. pneumophila sequences from the 3 patient samples were correctly identified as being highly related to their corresponding isolates (Fig. 6 and Fig. S5). However, the sequence quality and the breadth and depth of target coverage in the sample from patient 1P were superior to these parameters in samples from patients 3P and 5P. One explanation for this is the lower bacterial loads in the sputum samples of patients 3P and 5P, as indicated by the high *C_T_* values in Legionella PCR. Consistently, 3 further patient samples (2P, 4P, and 7P) contained very low bacterial loads and, not surprisingly, had insufficient coverage of targeted regions to allow phylogenetic analysis. This has been described elsewhere, such as in studies of direct whole-genome sequencing of Mycobacterium tuberculosis from respiratory specimens, which have shown that paucibacillary samples (as measured by smear or PCR) generated incomplete sequence coverage (17, 20).

Another challenge posed by sequencing of human respiratory specimens is the predominance of human DNA. Although FLASH-NGS has an additional advantage in blocking the sequencing of nontargeted DNA by dephosphorylation (19), a large proportion of the sequence reads in the patients were human DNA. One possible reason for this is the high concentration of starting DNA used in the patient samples, so that dephosphorylation may not have been optimal. Another contributing factor is the inevitable degree of human DNA shearing, which again disrupts the dephosphorylated DNA ends and allows them to be included in the final library. It remains to be determined whether improvements in sample quality, storage, extraction protocols, and optimization of methods of human DNA depletion could be of benefit (note that human DNA depletion by differential cell lysis has been used in sample-processing protocols for sequencing of M. tuberculosis from clinical specimens [16, 17]).

There are some limitations of the FLASH-NGS system. We found that four genes had low coverage for the bacterial isolates and the patient samples. Although it is unclear why these genes had lower coverage, they were all small genes, with only 2 to 3 target guides per gene. An improvement in the initial design of the target guides by including additional targets per gene and thus building redundancy may be beneficial. In addition, mutations in critical sites may affect gene coverage. In isolates 6 and 9, we found a mutation in the PAM motif (which is critical for enzyme cleavage) (Fig. S3), and this may be the cause of the low coverage in one gene. It would be of interest to assess how our method would perform on L. pneumophila isolates from other parts of the world. Another limitation for targeted NGS methods includes the current lack of user-friendly bioinformatics tools for sequence data analysis, particularly for the purposes of bacterial typing. Many software packages are currently geared toward analysis of whole-genome data or are proprietary, with high licensing fees making access difficult. This limited our FLASH-NGS data analysis to SNP-based phylogeny. The development of easy-to-access software packages for bioinformatics analysis of targeted NGS data will be important for the development and utility of this method.

### Conclusion.

This proof-of-concept study has demonstrated that phylogenetic analysis of L. pneumophila directly from respiratory samples is possible using targeted FLASH-NGS. Future studies that incorporate a larger number of patient samples will help to optimize and validate this method further and determine its utility in diagnostic laboratory and public health investigation settings.

## MATERIALS AND METHODS

An overview of the FLASH-NGS method used in this study is shown in the schematic in Fig. 1A. Briefly, genomic DNA extracted from samples is first dephosphorylated, thus blocking the DNA ends from adaptor attachment in the subsequent step of library preparation. The Cas9 enzyme is then led to specific locations within the L. pneumophila genome by a set of 20-bp CRISPR guide RNA molecules. At these target locations, Cas9 cleaves the DNA. The ends of the cleaved DNA molecules are not dephosphorylated and are therefore available for library preparation and attachment of adaptors. Amplification of the targeted regions and sequencing follows.

### Specimens.

The FLASH-NGS method was applied to 10 L. pneumophila isolates. This included 9 unique isolates from sporadic cases of legionellosis (all L. pneumophila PCR positive; 8 of 9 L. pneumophila [LP1] urine antigen positive) identified at New South Wales (NSW) Health Pathology Microbiology Laboratory Liverpool between 2016 and 2019 and one well-defined control isolate (L. pneumophila Philadelphia strain ATCC 33152). FLASH-NGS was also applied directly to six patient samples. These samples comprised endotracheal tube (ETT) aspirates (2 patients) and expectorated sputum (4 patients) and were the sources for L. pneumophila isolates 1, 2, 3, 4, 5, and 7 included in the culture arm of the study. A summary of the samples used in this study is included in Table 1.

### Storage of patient samples.

Excess respiratory specimens were stored at 4°C for up to 1 week during initial diagnostic investigations and subsequently frozen at −80°C for 4 to 21 months. The sample from patient 2P was stored as a DNA extract from sputum at −80°C for 42 months.

### Validation of the phylogenetic-analysis methods using a public data set of L. pneumophila genomes.

Publicly available whole-genome sequence data from 19 clinical and 2 control isolates of L. pneumophila that were part of several outbreak investigations in Sydney in 2016 (Table S1) (5) were used for the initial validation of the phylogenetic-analysis methods. The data were accessed via the NCBI Sequence Read Archive (SRA) under study accession number SRP117289.

### Ethics statement.

Ethics approval by the SWSLHD Human Research Ethics Committee was obtained for accessing patient data and excess stored respiratory specimens.

### L. pneumophila culture.

The 10 L. pneumophila culture isolates were grown on buffered charcoal-yeast extract (BCYE) medium (Thermo Fisher Scientific, Australia) at 37°C for 5 to 7 days.

### L. pneumophila PCR.

Legionella PCR was performed using the 16-well respiratory pathogens B assay, version 8 (AusDiagnostics, Mascot, NSW, Australia). The assay is a two-step nested multiplex tandem PCR with an initial round of 15 cycles, followed by a second round of 30 cycles. Results are reported as *C_T_* take-off values comprised of second-round PCR *C_T_* values plus 15 cycles. The assay differentiates between L. pneumophila and Legionella longbeachae.

### DNA extraction from L. pneumophila culture isolates.

Genomic DNA was extracted from 10 L. pneumophila isolates using the QIAamp DNA blood mini kit (Qiagen) with a 3-h proteinase K digest at 56°C. The final DNA extract was eluted in DNase-/RNase-free water, and the quality was measured using the NanoDrop ND-1000 (NanoDrop Technologies, Thermo Fisher Scientific, Australia). DNA purity was deemed to be satisfactory at a 260-nm/280-nm ratio of 1.6 to 2.2 and a 260-nm/230-nm ratio of >1.8. DNA concentration was measured by using the Qubit double-stranded DNA (dsDNA) HS assay kit (Thermo Fisher Scientific, Australia).

### DNA extraction from patient samples.

Endotracheal aspirate specimens from patients 1P and 7P and sputum specimens from patients 2P, 3P, 4P, and 5P were digested with Sputasol (ThermoFisher Scientific, Australia) at a 1:1 ratio and incubated for 10 min at 37°C, followed by centrifugation at 5,000 × *g* for 10 min. Genomic DNA was extracted using the QIAamp DNA blood mini kit (Qiagen, Hilden, Germany) with a 3-h proteinase K digest at 56°C. DNA was eluted in DNase-/RNase-free water, with quality and concentration measured as described above for L. pneumophila isolates.

### Target gene selection.

The 57 genes selected for targeted NGS in this study (Fig. S6A) were compiled from seven genes used for sequence-based typing (SBT) of L. pneumophila (21–23) and 50 genes used in a core-genome multilocus sequence typing (cgMLST) analysis (6).

### Guide RNA design and preparation.

A set of 293 guide RNAs were designed using the freely available FLASHit software (19), targeting the 57 genes of interest (Fig. S1A), with sequences derived from the reference genomic sequence of L. pneumophila Philadelphia (GenBank accession no. NC_002942.5). The FLASHit software removes from consideration any guides that match human DNA, allows zero mismatches in the protospacer-adjacent motif (PAM) proximal bases, and allows 2 or fewer mismatches in the full 20-mer (19). Characteristics of the guides are described in Fig. S6B and C. The 293 guide RNAs were synthesized by *in vitro* transcription, using the MEGAscript kit (Ambion Life Technologies, Australia), from a pool of single-stranded DNA oligonucleotides (Integrated DNA Technologies, Singapore) as previously described (19). The guide sequences and primers used to produce them are included in the supplemental material.

### WGS of L. pneumophila isolates.

Paired-end indexed libraries 100 bp in length were prepared from 1-ng purified DNA inputs with the Nextera DNA Flex library preparation kit (Illumina, Vic, Australia), according to the manufacturer’s instructions. DNA libraries were sequenced using the MiSeq system and the 2 × 100-bp kit, version 3 (Illumina, Victoria, Australia).

### FLASH-NGS of L. pneumophila isolates and patient samples.

Custom FLASH libraries were prepared from 25-ng DNA inputs for L. pneumophila isolates. For the patient samples, the DNA inputs were 100 ng for the sample from patient P1 and 12.5 to 30 ng for the samples from patients P2, P3, P4, P5, and P7. The smaller DNA inputs for patients P2 to P7 were limited by the overall low DNA concentration in these samples. The FLASH-NGS protocol has been described previously (19). Briefly, genomic DNA was blocked by phosphatase treatment with alkaline phosphatase (Sigma-Aldrich, Inc.) for 30 min at 37°C, followed by inactivation with sodium orthovanadate (New England Biolabs, Victoria, Australia). Target DNA was digested by Cas9 enzyme (Integrated DNA Technologies, Singapore) complexed to dual guide RNAs targeting the genes of interest for 2 h at 37°C, followed by inactivation with proteinase K (New England Biolabs, Victoria, Australia) and solid-phase reversible immobilization (SPRI) bead purification. Samples were given dAMP tails using the NEBNext dA-tailing module (New England Biolabs, Victoria, Australia), followed by adaptor ligation and indexing with 16 cycles of PCR according to the NEBNext Ultra II protocol (New England Biolabs, Victoria, Australia). Samples were SPRI bead purified, and the libraries were analyzed using the TapeStation system (Agilent Technologies, CA, USA). Libraries were quantified using the Qubit dsDNA HS assay kit (Thermo Fisher Scientific, Australia). Paired-end DNA libraries were sequenced using the MiSeq system and the 2 × 300-bp kit, version 3 (Illumina, Victoria, Australia).

### Analysis of L. pneumophila sequence data.

All raw sequence data were subjected to quality control procedures prior to further analysis. Demultiplexed reads were quality trimmed using Trimmomatic (24) (Illumina clip with sliding window of 4 and minimum read quality score of 20). Using FastQC (25) on trimmed samples showed mean quality scores above 30 for all samples sequenced by this study and above 25 for the public data set described in Table S1.

### L. pneumophila isolate WGS data analysis.

Reads were mapped to the reference genome of L. pneumophila Philadelphia (GenBank accession no. NC_002942.5) using the Burrows-Wheeler Aligner (BWA) (26) (Galaxy version 0.7.17.4). Mapping quality was assessed with QualiMap BamQC (27) (Galaxy version 2.2.2d+galaxy3). Base coverage over specific intervals (57 gene regions) was calculated from the number of nonduplicate reads aligned to each region and normalized for read length and target gene length. The numbers of nonduplicate reads aligned to specific regions were obtained using bedtools MultiCovBed (28) (Galaxy version 2.29.2).

For whole-genome assemblies, reads were assembled using SPAdes (29) (Galaxy version 3.12.0+galaxy1) and annotated with Prokka (30) (Galaxy version 1.14.5). The pangenome was calculated by using the Roary pipeline (31) (Galaxy version 3.13.0). The quality of the whole genomes was assessed using Quast (32) (Galaxy version 5.0.2+galaxy1).

Sequence types (STs) of L. pneumophila isolates were inferred from the assembled WGS data using legsta version 0.5.1 (33).

### Core-genome SNP analysis of WGS data.

Variant calling of paired-end reads was performed using Snippy (34) (Galaxy version 4.5.0) against the reference genome of L. pneumophila Philadelphia (GenBank accession no. NC_002942.5). Variant detection thresholds were set for a minimum coverage of 10, minimum mapping quality of 30, and minimum proportion of variant evidence of 0.75. Snippy outputs for the isolates were combined and analyzed by Snippy-core (34) (Galaxy version 4.5.0) to produce a core-genome single-nucleotide polymorphism (SNP) alignment.

### 57-gene SNP analysis of WGS data.

Variant calling was performed using Snippy (version 4.5.0) as described above. The SNPs within the 57 genes were extracted using SnpSift Intervals (35) (Galaxy version 4.3+t.galaxy0) and a browser extensible data (BED) file of the 57 gene regions. The variants were filtered for SNP variants only (excluding complex variants) using SnpSift Filter (35) (Galaxy version 4.3+t.galaxy0). A consensus sequence was created by applying variant call format (VCF) variants to the reference genome of L. pneumophila Philadelphia (NC_002942.5) using bcftools consensus (36) (Galaxy version 1.10). A FASTA file of all SNPs within the 57 genes was obtained using bedtools GetFastaBed (28) (Galaxy version 2.29.2) and a BED file containing the positions of all combined SNP variants from all isolates. The concatenated sequences of SNPs were aligned using MAFFT (37) (Galaxy version 7.221.3; fftns method). An overview of the bioinformatic workflow for 57-gene SNP-based phylogenetic analysis is provided in Fig. 1C.

Phylogenetic analysis was performed using the maximum-likelihood approach using MEGAX (version 10.1.8) and the general time-reversible (GTR) model with 500 bootstrap replicates.

### L. pneumophila isolate and patient FLASH-NGS data analysis.

For the L. pneumophila isolates, reads were mapped to the reference genome of L. pneumophila Philadelphia (GenBank accession no. NC_002942.5) using BWA-MEM (which seeds alignments with maximal exact matches) (38) (Galaxy version 0.7.17.1). Mapping quality, base coverage over specific intervals (57 gene regions), and SNP analysis of 57 targeted genes were performed as described above for WGS data analysis.

For the analysis of patient samples, FASTQ files were imported into CLC Genomic Workbench version 7.5.5 and mapped to the reference L. pneumophila Philadelphia genome sequence (NC_002942.5). Unmapped reads were discarded. Due to low coverage, the phylogenetic analysis was performed on fragments of 21 targeted genes. The coordinates for these gene fragments are included in Fig. S4. Variant calling was performed using Snippy (Galaxy version 4.5.0) against the reference genome sequence. Variant detection thresholds were set for a minimum coverage of 4, minimum mapping quality of 20, and minimum proportion of variant evidence of 0.75. The rest of the workflow for SNP analysis of the 21 gene fragments in patient samples and 10 L. pneumophila isolates was as described for the 57-gene SNP analysis for WGS data. SNPs were also assessed by manual inspection of the read alignment (.bam) files against the reference sequence of L. pneumophila (NC_002942.5) in Integrative Genomics Viewer (IGV; version 2.8.4).

For the 53-gene analysis of patient sample 1P and the 10 L. pneumophila isolates, the workflow was as described for the 57-gene SNP analysis for WGS data.

### Data availability.

Sequence data associated with this study have been deposited in the National Center for Biotechnology Information (NCBI) Sequence Read Archive (SRA). The BioProject accession number is PRJNA802331.
