# Supplementary material for: Culture-Free Phylogenetic Analysis of Legionella pneumophila Using Targeted CRISPR/Cas9 Next-Generation Sequencing
Source: Microbiol Spectr. 2022 Jul 11;10(4):e00359-22. doi: 10.1128/spectrum.00359-22 (PMC9430934; doi:10.1128/spectrum.00359-22)
Supplement: Supplemental file 1 — Supplemental material. Download spectrum.00359-22-s001.pdf, PDF file, 1.7 MB [file spectrum.00359-22-s001.pdf]

Supplemental Material

Culture-free phylogenetic analysis of *Legionella pneumophila* using targeted CRISPR/Cas9 Next Generation Sequencing

Ana Domazetovska*, Slade O. Jensen, Matthew Gray, Michael Radzieta, Michael Maley

*Corresponding author: Ana Domazetovska, Ana.Domazetovska@health.nsw.gov.au

TABLE S1. *Legionella pneumophila* serogroup 1 isolates from public dataset used for validation of the phylogenetic analysis methods in this study.

Isolate identification number	Source	Case number in outbreak	Association with location*	NCBI/SRA Accession number
RC2	Clinical	Case 5	Suburb 1	**SRX3177398**
RC1	Clinical	Case 5	Suburb 1	**SRX3177397**
RC3	Clinical	Case 4	Suburb 1	**SRX3177392**
BC3	Clinical	Case 7	CBD May	**SRX3177360**
BC4	Clinical	Case 7	CBD May	**SRX3177390**
BC8	Clinical	Case 8	CBD May	**SRX3177352**
BC6	Clinical	Case 6	CBD May	**SRX3177383**
GC4	Clinical	Case 12	Suburb 2/CBD May	**SRX3177384**
GC1	Clinical	Case 11	Suburb 2	**SRX3177351**
GC2	Clinical	Case 10	Suburb 2	**SRX3177382**
GC3	Clinical	Case 9	Suburb 2	**SRX3177381**
Reference 2013	Clinical	Reference case 1	Not related	**SRX3177362**
Reference 2015	Clinical	Reference case 2	Not related	**SRX3177361**
BC1	Clinical	Case 1	CBD March	**SRX3177358**
BC2	Clinical	Case 1	CBD March	**SRX3177359**
BC7	Clinical	Case 1	CBD March	**SRX3177356**
BC5	Clinical	Case 3	CBD March	**SRX3177354**
BC9	Clinical	Case 2	CBD March	**SRX3177357**
BC10	Clinical	Case 2	CBD March	**SRX3177355**
Reference control 1	ATCC33152			**SRX3177353**
Reference control 2	ATCC 33215			**SRX3177386**

* CBD March, isolates from Sydney Central Business District February to March 2016; CBD May, isolates from Sydney Central Business District April to May 2016. Sequence data generated by Timms *et al*. 2018 (1).

NCBI = National Centre for Biotechnology Information; SRA = Sequence Read Archive

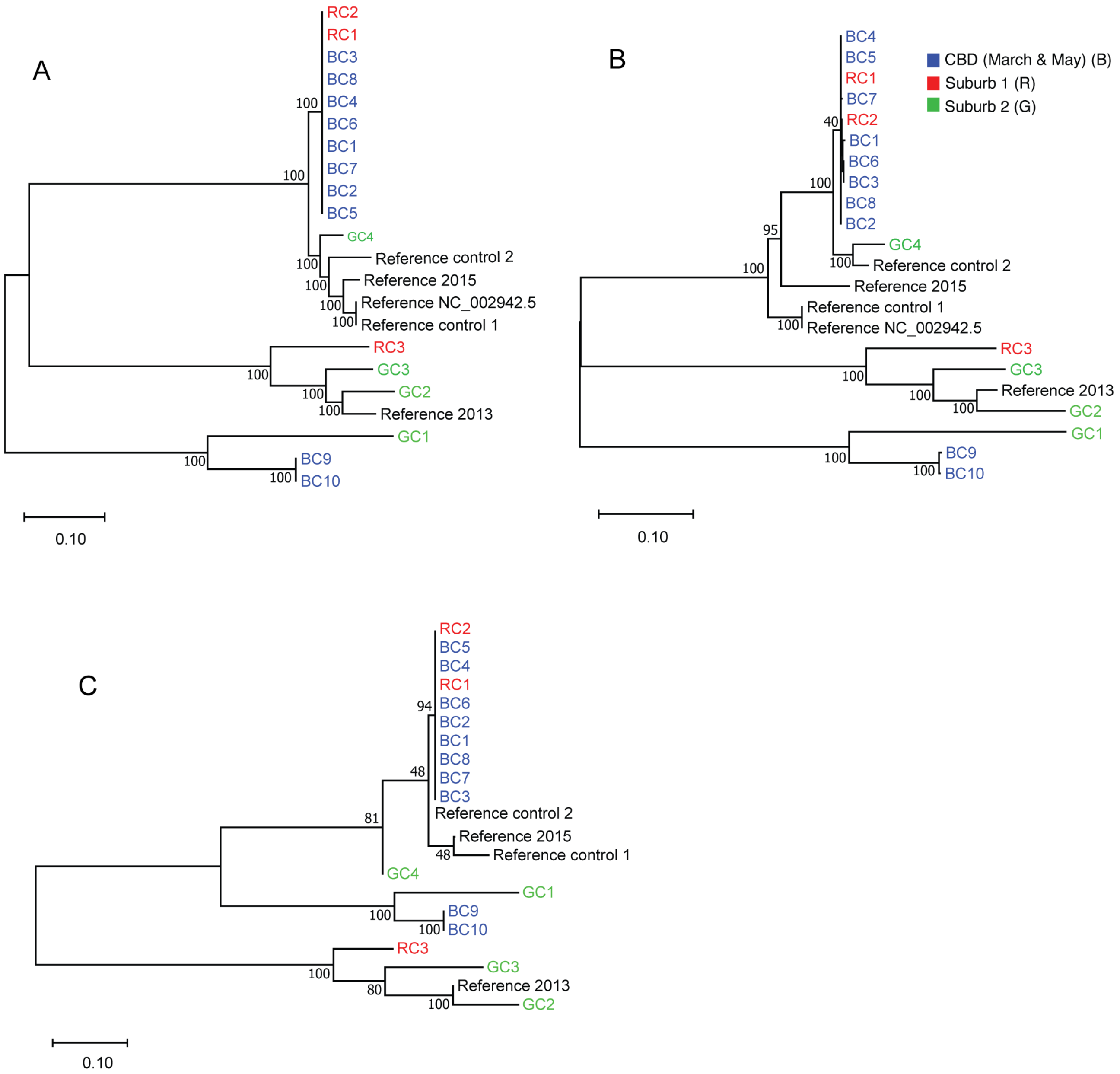

FIG S1. Validation of phylogenetic analysis methods on a public dataset. **(A)** Core SNP-based mapping phylogeny of outbreak *L. pneumophila* isolates between February and May 2016 in Sydney. A maximum likelihood phylogenetic tree based on 133,958 core SNPs relative to reference *L. pneumophila* Philadelphia (NC_002942.5). The method correctly identified the outbreak cluster compared on previously published phylogeny (1). The main cluster contains isolates from cases of Central Business District (CBD) March; case 1 (BC1, BC2 and BC7) and case 3 (BC5); suburb 1, case 5 (RC1 and RC2); CBD May, cases 6 (BC6), 7 (BC3 and BC4), and 8 (BC8). **(B)** 57 gene SNP-based mapping phylogeny of the same samples produced congruent results. Maximum likelihood phylogenetic tree based on 2,126 SNPs within 57 genes. **(C)** 21 gene fragment SNP-based mapping phylogeny of the same samples produced congruent results. Maximum likelihood phylogenetic tree based on 343 SNPs within 21 genes.

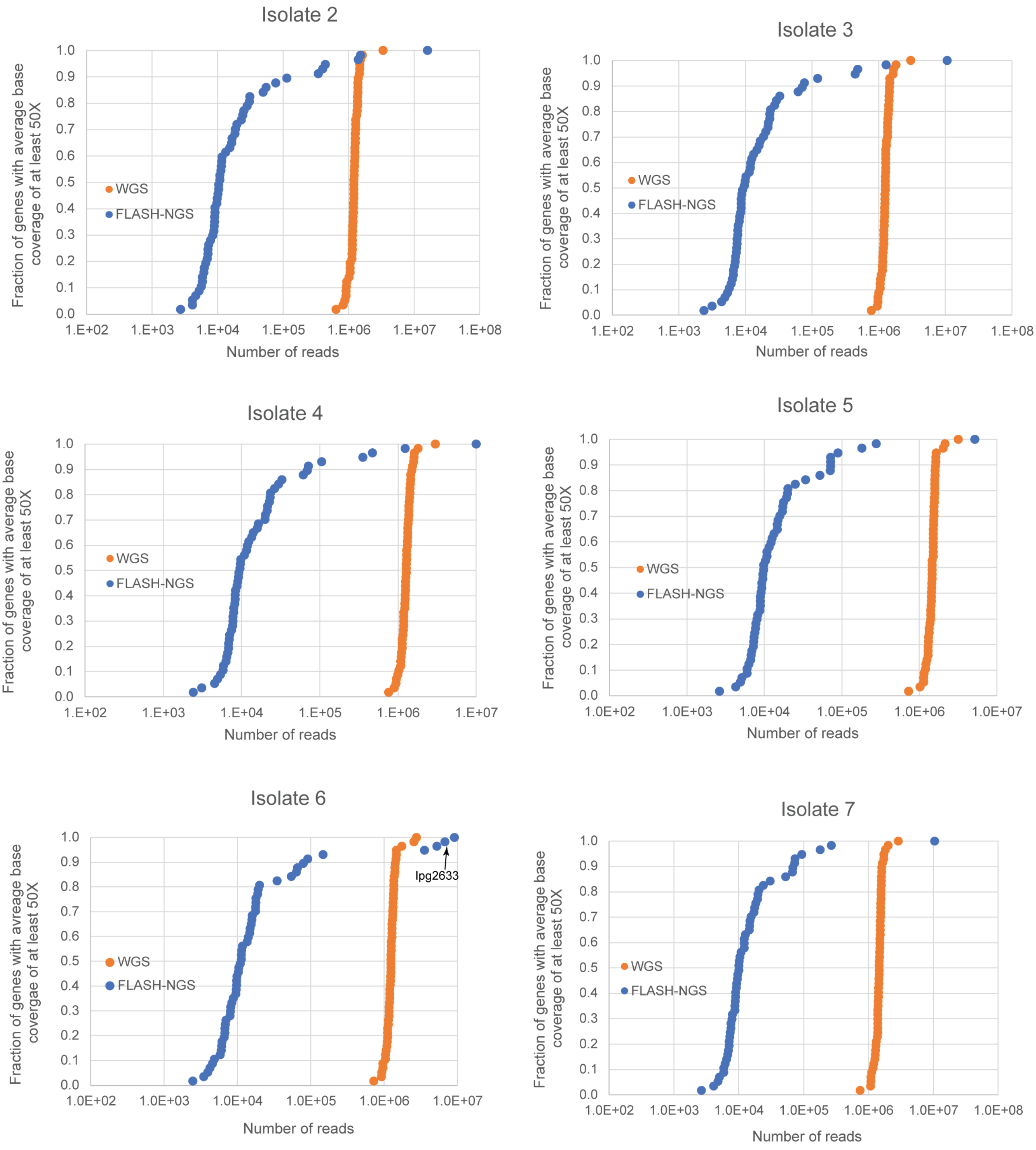

FIG S2. See next page for figure legend.

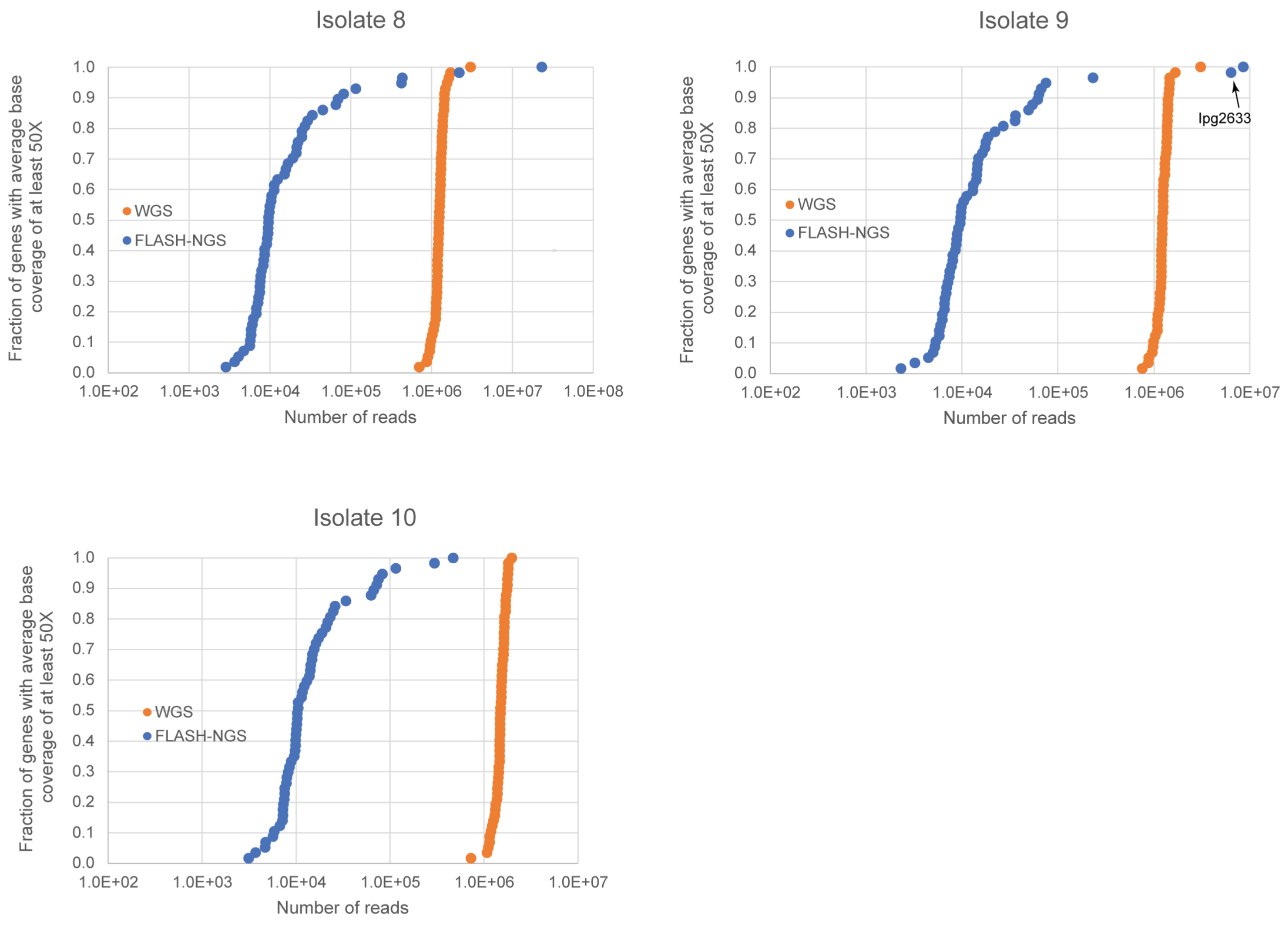

FIG S2. FLASH-NGS compared to WGS on cultured *L. pneumophila* isolates. Number of reads needed to achieve per base coverage of at least 50X per gene target. Results for isolates 2-10 are shown. Each data point represents an individual gene sequenced by FLASH-NGS (blue) and WGS (orange).

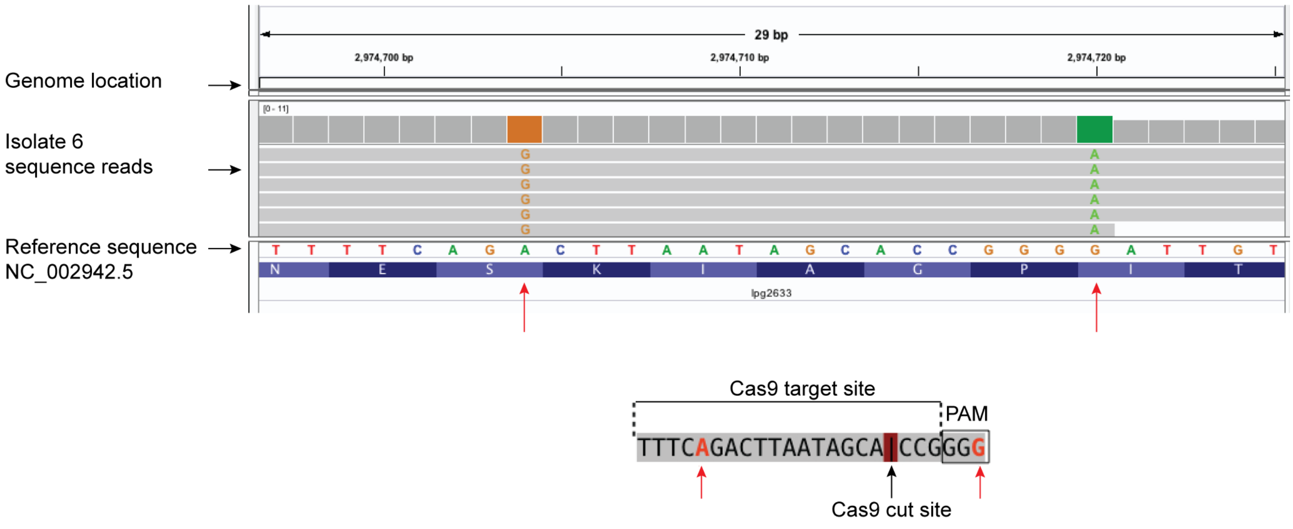

FIG S3. Mutations in the CRISPR/Cas9 target site are likely cause for low coverage of gene lpg2633 in Isolates 6 and 9. Image shows 2 mutations (red arrows), one located within the Cas 9 target site (A to G) and other (G to A) located within the proto-spacer adjacent motif (PAM) which flanks the target site and is essential for enzyme cleavage to occur. These 2 mutations were only present within the lpg2633 sequence of *L. pneumophila* isolates 6 and 9.

	Gene	Coordinates (bp)	
1	NC_002942.5_lpg0136|pykA	163386	163822
		164068	164524
2	NC_002942.5_lpg0245	289686	289904
		290113	290412
		290767	290999
3	NC_002942.5_lpg0419|glk	459385	459644
		459847	460093
4	NC_002942.5_lpg0467|proA	509531	509731
		510150	510360
5	NC_002942.5_lpg0601|ycf24	633980	634181
6	NC_002942.5_lpg0607	639708	639911
7	NC_002942.5_lpg0622	655961	656204
8	NC_002942.5_lpg0628	663612	663811
9	NC_002942.5_lpg0890|MetC	962876	963092
		963484	963697
10	NC_002942.5_fliC|flaA	1479119	1479339
11	NC_002942.5_lpg1503|aceF	1662751	1662958
12	NC_002942.5_lpg1811|lysC	2024749	2024958
		2026613	2026850
13	NC_002942.5_lpg1909	2129877	2130460
14	NC_002942.5_lpg2229	2521182	2521434
15	NC_002942.5_asd	2603951	2604239
16	NC_002942.5_lpg2331|bioC	2634676	2635130
		2635367	2635612
17	NC_002942.5_lpg2654	3000321	3000738
		3001128	3001355
18	NC_002942.5_lpg2691|momp	3042685	3043163
		3043380	3043602
		3044314	3044515
19	NC_002942.5_lpg2864	3240892	3241091
		3241495	3241698
20	NC_002942.5_lpg2882|metG	3261823	3262293
		3263560	3263787
21	NC_002942.5_lpg2961	3351608	3351809

FIG S4. List of selected 34 fragments within 21 targeted genes in *L. pneumophila* used in phylogenetic analysis of patient samples. The gene sequences were derived from reference *L. pneumophila* Philadelphia (GenBank accession NC_002942.5).

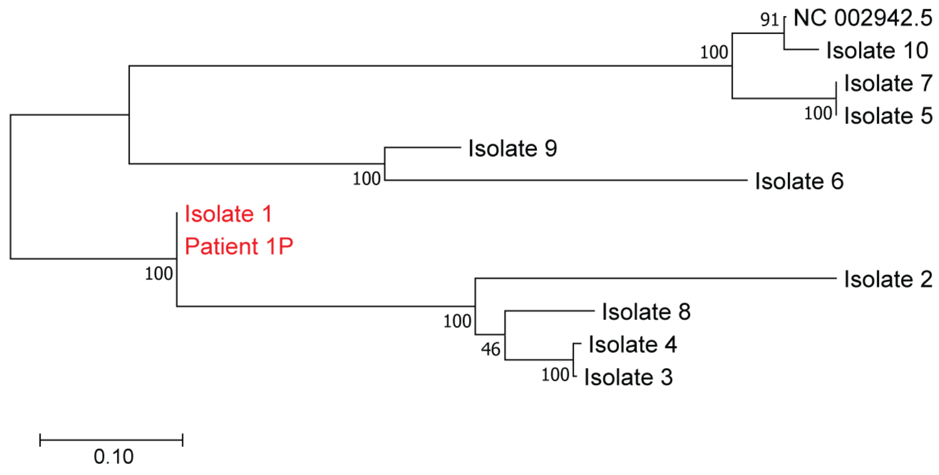

FIG S5. Phylogenetic analysis of FLASH-NGS data. 53 gene SNP-based mapping phylogeny on the 10 *L. pneumophila* isolates and patient 1P. Four genes were excluded from the analysis due to low coverage in patient 1P. Maximum likelihood phylogenetic tree based on 1535 SNPs within 53 genes relative to the reference.

A

NC_002942.5_lpg0189
NC_002942.5_lpg0890|Metc
NC_002942.5_lpg2528
NC_002942.5_lpg0104
NC_002942.5_lpg2669
NC_002942.5_lpg2882|metG
NC_002942.5_lpg0689
NC_002942.5_fliC|flaA
NC_002942.5_lpg2494
NC_002942.5_lpg2264
NC_002942.5_lpg2691
NC_002942.5_lpg1586
NC_002942.5_lpg0622
NC_002942.5_lpg1737|gatB
NC_002942.5_lpg2331|bioC
NC_002942.5_lpg2349
NC_002942.5_lpg0419|glk
NC_002942.5_lpg2864
NC_002942.5_lpg1503|aceF
NC_002942.5_lpg0871
NC_002942.5_lpg0329|rplC
NC_002942.5_lpg0812|mreC
NC_002942.5_lpg2902
NC_002942.5_lpg2229
NC_002942.5_lpg0136|pykA
NC_002942.5_lpg2654
NC_002942.5_lpg0409
NC_002942.5_lpg2878|corA
NC_002942.5_lpg2387|pla
NC_002942.5_lpg0085
NC_002942.5_lpg2961|momp
NC_002942.5_lpg0664|rpe
NC_002942.5_lpg1323
NC_002942.5_lpg1869|rnc
NC_002942.5_lpg0628
NC_002942.5_lpg2633
NC_002942.5_asd
NC_002942.5_lpg0525
NC_002942.5_lpg0866
NC_002942.5_lpg1543
NC_002942.5_lpg2597
NC_002942.5_lpg1909
NC_002942.5_lpg0245
NC_002942.5_lpg0957
NC_002942.5_neuA
NC_002942.5_lpg1811|lysC
NC_002942.5_lpg0331|rplW
NC_002942.5_lpg0601|ycf24
NC_002942.5_lpg1534|hemL
NC_002942.5_lpg0467|proA
NC_002942.5_lpg0607
NC_002942.5_lpg1744|ydiC
NC_002942.5_Mip
NC_002942.5_lpg0596
NC_002942.5_lpg0131|dapB
NC_002942.5_lpg1759|fliG
NC_002942.5_lpg0700|pcm

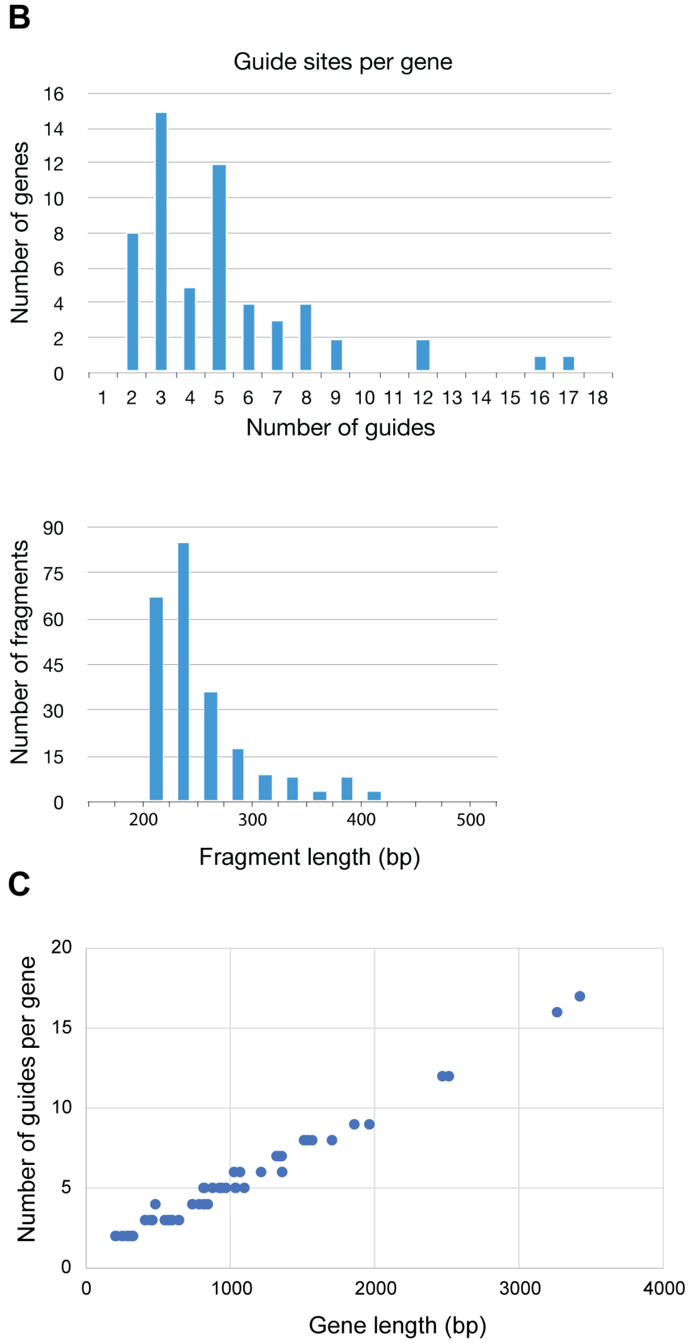

FIG S6. (A) List of the 57 targeted genes in *L. pneumophila*. The gene sequences were derived from reference *L. pneumophila* Philadelphia (GenBank accession NC_002942.5) **(B)** Histograms showing properties of the 293 guide RNA set designed to target 57 *L. pneumophila* genes. **(C)** The number of guides per gene has a linear relationship with gene length. Each data point represents one of 57 target genes.

REFERENCES

1. Timms VJ, Rockett R, Bachmann NL, Martinez E, Wang Q, Chen SC, Jeoffreys N, Howard PJ, Smith A, Adamson S, Gilmour R, Sheppeard V, Sintchenko V. 2018. Genome Sequencing Links Persistent Outbreak of Legionellosis in Sydney (New South Wales, Australia) to an Emerging Clone of Legionella pneumophila Sequence Type 211. Appl Environ Microbiol 84:e02020-17.
